# Distribution of serum neuron‐specific enolase and the establishment of a population reference interval in healthy adults

**DOI:** 10.1002/jcla.22863

**Published:** 2019-02-19

**Authors:** Qian Liu, Jilong Fan, Aiguo Xu, Li Yao, Yan Li, Wenjun Wang, Wei Liang, Fumeng Yang

**Affiliations:** ^1^ Department of Laboratory Medicine The Second People's Hospital of Lianyungang Lianyungang China; ^2^ Department of Hepatobiliary Surgery The Second People's Hospital of Lianyungang Lianyungang China; ^3^ Department of Oncology The Second People's Hospital of Lianyungang Lianyungang China

**Keywords:** neuron‐specific enolase, population reference interval, serum

## Abstract

**Background:**

Neuron‐specific enolase (NSE) is an important tumor marker in the serum of patients with lung cancer. Elevated serum NSE levels are also associated with many other diseases. However, there is no unified population reference interval for serum NSE. This study aimed to investigate the distribution of serum NSE in healthy Chinese adults aged 20‐79 years and to establish its reference interval in Chinese population.

**Methods:**

A total of 10 575 healthy subjects were in line with the requirements of this study. The concentration of serum NSE was detected by a fully automated Cobas e602 analyzer with matching reagents. The population reference interval for serum NSE was established using the unilateral 95th percentile (*P*_95_) according to standard guidelines.

**Results:**

The distributions of serum NSE were not significantly different between males and females (*P* > 0.05) and also did not differ by age (*P* > 0.05). Therefore, the population reference interval for serum NSE was established as upper limit 25.4 ng/mL (90% confidence interval: 24.5‐26.2 ng/mL).

**Conclusions:**

We established the first population reference interval for serum NSE in a large healthy Chinese adult cohort, which was higher than that recommended by Roche Diagnostics GmbH. This new reference interval is more practical and applicable in Chinese adults.

**Abbreviations**
AFP: alpha‐fetoprotein
CA199: carbohydrate antigen 199
CEA: carcinoembryonic antigen
CI: confidence interval
FBG: fasting blood glucose
IQR: interquartile range
NSE: neuron‐specific enolase
TC: total cholesterol
TG: triglycerides

## 1 INTRODUCTION

Lung cancer is the most commonly diagnosed cancer worldwide (11.6% of all cancer cases) and the leading cause of cancer death (18.4% of all cancer deaths).^1^ Lung cancer can be divided into two main types: non–small‐cell lung cancer and small‐cell lung cancer. About 80%‐85% of lung cancers are non–small‐cell lung cancer.^2^ Neuron‐specific enolase (NSE) is an important tumor marker in the serum of patients with lung cancer.^3^, ^4^, ^5^ Elevated serum NSE levels are also associated with neuroblastoma, neuroendocrine neoplasms, renal cell carcinoma, multiple myeloma, brain trauma, Guillain‐Barre syndrome, and other diseases.^6^, ^7^, ^8^, ^9^ Therefore, measurement of serum NSE can be used as an auxiliary marker for the diagnosis of several diseases. Currently, however, there is no unified population reference interval for serum NSE. Independent clinical laboratories often refer to the reagent manufacturer's instructions for the population reference interval for serum NSE. This interval has not been validated, however, and therefore, the utility of serum NSE is limited in clinical applications.^10^ Moreover, the tested level of serum NSE will also be affected by detection system, genotype, geographical location, lifestyle, and many other factors.^11^ Thus, the population reference interval of serum NSE could be variable in different population. However, most Chinese laboratories use the NSE population reference intervals either provided by aboard (Europe and the United States) laboratories or the reagent manual.^11^ These population reference intervals may not be suitable for Chinese population in clinical application. In order to establish a more specific and reliable population reference interval of serum NSE for Chinese population, we measured serum NSE in a large number of healthy adult subjects aged 20‐79 years and established the population reference interval of serum NSE according to Clinical and Laboratory Standards Institute (CLSI) CA28‐A3.^12^

## 2 MATERIAL AND METHODS

### 2.1 Study subjects

**Figure 1:**
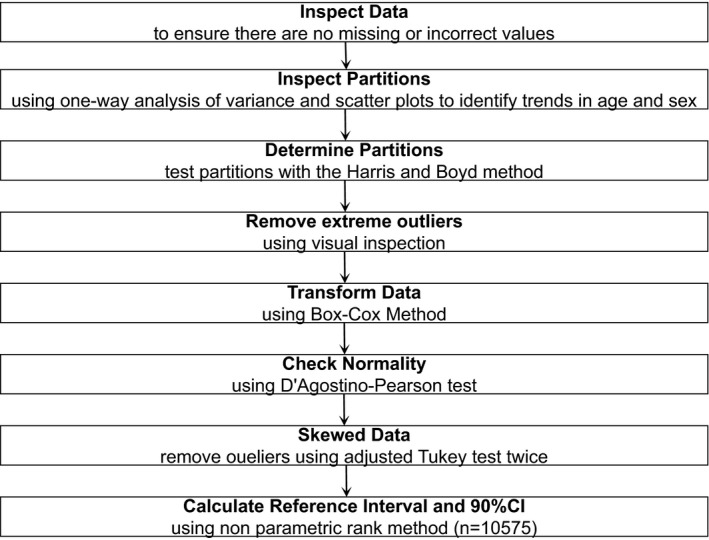
Establishing reference interval of serum NSE on the bias of CLSI CA28‐A3

A total of 11 000 subjects who completed a physical examination at the Physical Examination Center of the Second People's Hospital of Lianyungang from January 2017 to June 2018 were randomly selected as participants in this study. According to the inclusion and exclusion criteria, a total of 10 575 healthy individuals (5056 males and 5519 females; 20‐79 years old) were in line with the requirements of the study. Participants were divided into 12 groups by sex (male and female) and age (20‐29 years old; 30‐39 years old; 40‐49 years old; 50‐59 years old; 60‐69 years old; and 70‐79 years old) as recommended by CLSI C28‐A3 to determine whether the distribution of serum NSE levels differs by age and/or sex.^12^ The detailed screening procedures of the study participants are shown in Figure 1.

Participants in this study met the following inclusion criteria: (a) 20‐79 years old; (b) blood pressure ≤139/89 mm Hg; (c) serum indices of samples were all negative (means no hemolysis, no icterus, and no lipemia); (d) routine biochemical indicators, such as serum urea, creatinine, total cholesterol, triglycerides, and fasting blood glucose, within the normal reference intervals; (e) negative for the presence of indicators of infectious diseases, such as hepatitis B, hepatitis C, and syphilis caused by *Treponema pallidum*; and (f) indicators for various tumors markers, such as CYFRA21‐1, CA199, CEA, and AFP, within normal reference intervals (Table 1). Participants were excluded if they had (a) acute inflammation or infection; (b) acute or chronic liver, kidney, lung, brain, heart, or other systemic disease; (c) a history of surgery within the past 6 months; (d) a history of drug treatment in the past month; (e) recent diet; or (f) recent irregular work schedules, insufficient sleep, or excessive alcohol consumption. This study was approved by the Medical Ethics Committee of the Second People's Hospital of Lianyungang. Written informed consent was obtained from all participants.

**Table 1:** Normal reference interval of routine indicators

Parameters	Reference interval	Units of measurement
Urea	3.1‐8.0 (male, ages 20‐59)	mmol/L
	3.6‐9.5 (male, ages 60‐79)	
	2.6‐7.5 (female, ages 20‐59)	
	3.1‐8.8 (female, ages 60‐79)	
Creatinine	57‐97 (male, ages 20‐59)	μmol/L
	57‐111 (male, ages 60‐79)	
	41‐73 (female, ages 20‐59)	
	41‐81 (female, ages 60‐79)	
TC	<200	mg/dL
TG	<150	mg/dL
FBG	70‐110	mg/dL
Hepatitis B	Negative	—
Hepatitis C	Negative	—
Syphilis	Negative	—
CYFRA21‐1	<3.3	ng/mL
CA199	<27	U/mL
CEA	<4.7	ng/mL
AFP	<7.0	ng/mL

AFP, alpha fetoprotein; CA199, carbohydrate antigen 199; CEA, carcinoembryonic antigen; FBG, fasting blood glucose; TC, total cholesterol; TG, triglycerides.

### 2.2 Specimen collection

Five milliliters of fasting venous blood was collected in the morning. The samples were centrifuged within 2 hours, and packed serum was stored at −80°C until testing. Ten milliliters of fresh morning urine also was collected. Routine urine measurements were completed within 2 hours of collection.

### 2.3 Instruments and reagents

To minimize analytical errors, the same lots of reagents, standards, and quality control materials (means to check system with traditional QC runs which helps detect both bias shifts and imprecision) were used throughout the analyses. Serum NSE levels were analyzed via electrochemiluminescence immunoassay (ECLIA) using a standard assay kit for in vitro diagnostics (NSE kit lot: 28293601; NSE calibrator lot: 27153901; Roche Diagnostics GmbH, Mannheim, Germany). Reactions and quantitation were performed using a fully automated Cobas e602 analyzer (Roche Diagnostics GmbH). Tumor marker substance controls (lot 1: 256628; lot2: 256629; Roche Diagnostics GmbH) were used to ensure the precision of the test results. Urinalysis was conducted using an HC900 automatic urine analyzer (Changchun Dirui Co., Ltd, Changchun, China). All instruments and equipment were maintained and calibrated according to the manufacturer's instructions, and the indoor quality control and inter‐room quality evaluation met all requirements prior to specimen evaluation.

### 2.4 Statistical analyses

#### 2.4.1 Outlier test

According to the Dixon method recommended in the CLSI C28‐A3 guidelines,^12^ the detection result is sorted by size to calculate the range, R. The distribution then is used to calculate the difference (D) between the maximum value and the minimum value and its adjacent value. If D/R ≥ 1/3, then the maximum or minimum value is treated as an outlier. This process is repeated on the remaining data until all outliers are eliminated.

#### 2.4.2 Identification of biological reference interval groups

We referred to the recommended methods in CLSI C28‐A3 and WS/T 402‐2012 ("Establishment of reference interval for clinical laboratory test items"),^12^, ^13^ and applied the *Z* test to determine whether the data from two comparison groups can be combined. The formula for the *Z* test is:

$$Z=\frac{\bar{x_{1}}-\bar{x_{2}}}{\sqrt{\frac{s_{1}^{2}}{n_{1}}+\frac{s_{2}^{2}}{n_{2}}}},\;Z^{*}\;=\;\sqrt[{3}]{\frac{n_{1}\;+\;n_{2}}{240}}$$

Where ${\overset{¯}{x}}_{1}$ and ${\overset{¯}{x}}_{2}$ represent the mean values from the two groups, *s*_1_ and *s*_2_ represent the standard deviations from the two groups, and *n*_1_ and *n*_2_ represent the sample sizes of the two groups. If *Z* > Z*, then the difference between the reference intervals is statistically significant (*P* < 0.05) and the reference intervals for the two groups should remain separate. In contrast, if *Z* < Z*, then the difference between the two reference intervals is not statistically significant (*P* > 0.05), and the intervals can be merged into one interval.

#### 2.4.3 Statistical analysis

The data were analyzed in IBM SPSS Statistics version 20 (Armonk, NY, USA). The Kolmogorov‐Smirnov test was applied to test the normality of the data. Non‐normal data were expressed using the median and interquartile range (IQR), and the Kruskal‐Wallis H test was used to compare across multiple groups. Non‐parametric (ranked) method was used to establish reference interval by CLSI C28‐A3. A *P*‐value <0.05 was considered statistically significant.

## 3 RESULTS

**Figure 2:**
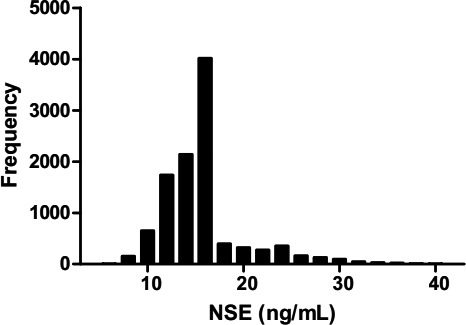
Distribution histogram of serum neuron‐specific enolase (NSE) levels

A total of 10 575 individuals were ultimately retained for inclusion in this study, including 5056 males and 5519 females. Kolmogorov‐Smirnov test showed a skewed distribution for serum NSE level (*P* < 0.05, Figure 2). The distribution of serum NSE levels was not significantly different between males and females, which indicated that the levels of NSE were not correlated with gender (*Z* = 4.89, Z* = 19.91, *P* > 0.05; Table 2). Furthermore, no significant age‐related differences in serum NSE levels were observed, which showed that the levels of NSE were not correlated with age (*P* > 0.05; Table 3).

**Table 2:** Distribution of serum neuron‐specific enolase (NSE) by sex

Gender	n	Median (IQR)	Upper limit	*Z* test
Male	5056	15.3 (13.8‐15.9)	25.5 (90% CI: 24.7‐26.5)	*Z* < *Z* ^*^ *P* > 0.05
Female	5519	14.7 (12.4‐15.9)	24.8 (90% CI: 23.9‐25.8)	
Total	10 575	15.3 (13.8‐15.9)	25.4 (90% CI: 24.5‐26.2)	

CI, confidence interval; IQR, interquartile range.

The unit of NSE is ng/mL.

**Table 3:** Distribution of serum neuron‐specific enolase (NSE) by age

Age group	n	Median (IQR)	Upper limit	*H*‐value	*P*‐value
20‐29 (male)	224	15.2 (13.7‐15.8)	24.1 (90% CI: 20.2‐29.2)	7.66	0.18
30‐39 (male)	743	15.3 (13.7‐15.9)	24.1 (90% CI: 21.3‐26.0)		
40‐49 (male)	1235	15.4 (14.2‐15.9)	26.8 (90% CI: 24.0‐27.9)		
50‐59 (male)	1248	15.3 (13.9‐15.9)	24.9 (90% CI: 23.9‐26.4)		
60‐69 (male)	905	15.3 (13.7‐15.9)	25.1 (90% CI: 23.3‐27.0)		
70‐79 (male)	701	15.3 (13.7‐15.9)	24.7 (90% CI: 23.4‐25.8)		
20‐29 (female)	351	15.1 (13.1‐16.3)	24.6 (90% CI: 23.8‐25.8)	9.27	0.10
30‐39 (female)	809	14.5 (12.2‐16.0)	26.4 (90% CI: 24.8‐27.7)		
40‐49 (female)	1350	14.7 (12.4‐16.2)	24.6 (90% CI: 23.9‐25.4)		
50‐59 (female)	1143	14.8 (12.5‐16.3)	26.4 (90% CI: 24.6‐27.7)		
60‐69 (female)	1021	14.5 (12.2‐15.9)	20.8 (90% CI: 17.2‐26.4)		
70‐79 (female)	845	14.9 (12.6‐15.8)	20.8 (90% CI: 17.3‐26.4)		

IQR, interquartile range.

The *H*‐value is the approximate χ^2^ value.

The unit of NSE is ng/mL.

In accordance with non‐parametric method recommended by CLSI C28‐A3 and WS/T 402‐2012, the upper reference limit is important, which was set at the 95th percentile value. Accordingly, the population reference interval for serum NSE was established as upper limit 25.4 ng/mL by using the method of ECLIA in this study.

## 4 DISCUSSION

As an important tumor marker, serum NSE has been widely clinically used in the world.^14^, ^15^ Just like other markers, a measurement result of serum NSE by itself without any appropriate reference interval or medical decision limit is of little value. The reference interval is essential for the interpretation of clinical laboratory results and patient care.^16^ Thus, the development of an appropriate reference interval can provide an important basis for the diagnosis, progression, and prognosis of clinical diseases.^17^ However, the establishment of reference interval is constrained and influenced by many factors, such as ethnic characteristics and living environment.^18^, ^19^ And regrettably, there have been only little reports about the establishment of serum NSE reference interval in healthy population up to date. Therefore, this is, to the best of our knowledge, the first study to establish the reference interval of serum NSE by using ECLIA in a large sample size of healthy Chinese population.

In this study, all subjects were rigorously screened, and 10 575 participants met the requirements to be included in the construction of the serum NSE reference interval. Rigorous statistical analyses were applied to show that the distribution of serum NSE levels was not significantly different between males and females (*P* > 0.05). In a similar study, Wen et al conducted a serum NSE reference interval study among healthy adults ranging in age from 17 to 62 years and reported no sex differences in serum NSE levels,^20^ which is consistent with our findings. In contrast, Li et al^21^ reported significant differences in serum NSE levels by sex and established separate reference intervals for males and females. Equivocal findings across studies may be due to sampling error, different regional characteristics, differences between individuals, or different detection methods.

The present study followed the recommendations set forth by the CLSI C28‐A3 to examine interval ranges by age.^12^ No significant differences in serum NSE levels were observed in our participant cohort (*P* > 0.05). This finding is consistent with a previous study by Guo et al^22^, which reported no differences in serum NSE levels across 148 healthy adults aged 17‐85 years. These previous findings are therefore consistent with the results of our study and support our determination of a common population reference interval for serum NSE levels for males and females between the ages of 20 and 79 years old.

This study established a serum NSE population reference interval of upper limit 25.4 ng/mL (90% confidence interval: 24.5‐26.2), which is significantly higher than the reference interval stated by Roche Diagnostics GmbH. The upper limit of the reference interval provided by the Roche NSE kit is 17.0 ng/mL.^23^ This difference may be due to population disparities and inter‐individual variation in the cohort used to construct the interval. Approximately 20% of healthy individuals exhibited elevated NSE levels based on the reference interval provided by the kit, even though all other indicators were normal. Our findings indicate, however, that the upper limit of the kit's reference interval is too low. Under the new reference interval established in our study, these individuals would fall within the normal reference interval, significantly reducing false‐positive results.

In summary, we established the reference interval for serum NSE using ECLIA in large‐scale healthy Chinese population. Furthermore, the findings from this study suggest that every laboratory should establish a serum NSE population reference interval that accounts for the regional population, factors in the regional environment, and detection system characteristics.

## AUTHORS’ CONTRIBUTIONS

QL and FY conceived and designed the experiments. QL, JF, AX, and LY performed the experiments. YL, WW, and WL analyzed the data. QL, JF, and FY wrote the paper.
